# Estrus Detection and Dairy Cow Identification with Cascade Deep Learning for Augmented Reality-Ready Livestock Farming

**DOI:** 10.3390/s23249795

**Published:** 2023-12-13

**Authors:** İbrahim Arıkan, Tolga Ayav, Ahmet Çağdaş Seçkin, Fatih Soygazi

**Affiliations:** 1 Computer Engineering Department, İzmir Institute of Technology, Izmir 35430, Türkiye; ibrahimarikan064@gmail.com (İ.A.); tolgaayav@iyte.edu.tr (T.A.); 2 Computer Engineering Department, Aydın Adnan Menderes University, Aydın 09100, Türkiye; acseckin@adu.edu.tr

**Keywords:** artificial intelligence, augmented reality, dairy cow identification, deep learning, estrus detection, image processing, livestock, precision livestock farming, transfer learning

## Abstract

Accurate prediction of the estrus period is crucial for optimizing insemination efficiency and reducing costs in animal husbandry, a vital sector for global food production. Precise estrus period determination is essential to avoid economic losses, such as milk production reductions, delayed calf births, and disqualification from government support. The proposed method integrates estrus period detection with cow identification using augmented reality (AR). It initiates deep learning-based mounting detection, followed by identifying the mounting region of interest (ROI) using YOLOv5. The ROI is then cropped with padding, and cow ID detection is executed using YOLOv5 on the cropped ROI. The system subsequently records the identified cow IDs. The proposed system accurately detects mounting behavior with 99% accuracy, identifies the ROI where mounting occurs with 98% accuracy, and detects the mounting couple with 94% accuracy. The high success of all operations with the proposed system demonstrates its potential contribution to AR and artificial intelligence applications in livestock farming.

## 1. Introduction

In today’s context, agricultural and livestock sectors make significant changes in order to increase labor productivity and become more efficient [1,2,3]. Augmented reality (AR) technology is gaining increasing importance for the success of precision agriculture. Emerging technologies, such as data-driven farming and autonomous agricultural robots, provide substantial advantages in terms of data visualization, animal monitoring, and access to information, suggesting that this technology may find broader applications in agriculture and food supply chain domains in the future [4]. For instance, Caria and others have emphasized the significance of AR technology in the context of precision livestock farming, highlighting its crucial role in enabling the real-time monitoring of animals during farm operations [5]. Augmented reality can enhance farm management by providing farmers with real-time access to details, such as milking, feeding, and breeding of animals, thereby improving efficiency and accuracy in farm operations. Particularly, AR-based smart glasses can display information, like animal identification numbers, health status, genetic characteristics, and production data, making the process of animal selection and management more efficient and precise, thus offering substantial advantages to farmers [6]. AR technologies can also assist farmers in navigation and guidance, especially in large-scale farms. They can be utilized to determine the locations of animals and facilities using GPS and sensors. For example, AR-based smart glasses can provide directions to specific animals or groups of animals, as well as suggest the most optimal routes to reach them, which can reduce the time and effort required for animal tracking and grouping [7]. Another example of the use of AR in the field of livestock is its application in improving the education and training of veterinary students and professionals. AR can provide interactive learning experiences by simulating animal anatomy, physiology, diseases, and treatments [8,9].

Industry 4.0 and precision livestock farming (PLF) have enabled a modern technological approach to animal farming and production, encompassing ethical, economic, and logistical aspects [10]. The advent of Industry 4.0 and the Internet of Things (IoT) have enabled the continued advancement and development of PLF. Everyday farming practices coupled with continuous and real-time monitoring of animal parameters can have significant impacts on welfare and health assessment. The term Agriculture 4.0 emerged from the term Industry 4.0. However, the benefits that Industry 4.0 bring to industrial use cases may not be fully transferable to livestock farming [11]. The presence of individual living animals and the strong environmental impact of livestock farming affect the role of digital individualization and demand orientation. The introduction and adoption of Industry 4.0 concepts and technologies may contribute significantly to transforming agriculture into something that may be called Agriculture 4.0.

AR is still an emerging technology in the field of agriculture [4]. It can potentially help farmers with training by providing an interactive and safe form of training. However, its usage in agriculture is unexplored. AR technologies are employed in agriculture and livestock to enhance data visualization, integrate with Industry 4.0 technologies, support disease detection, facilitate real-time monitoring, and enable individual animal management, as well as improve efficient access to information and animal tracking. These applications aim to enhance overall efficiency, productivity, and sustainability in the agricultural sector. Factors such as monitoring, identification, and estrus detection in animals hold significant importance in dairy cow farming, both in open and closed environments. These processes facilitate the close monitoring of animal health, early disease detection, and timely treatment when needed. Furthermore, animal identification allows for accurate record keeping and data tracking, thereby enhancing efficiency. Estrus detection, when accurately timed, improves reproductive efficiency and enables more effective management of genetic resources. Therefore, animal monitoring and management play a critical role in both animal welfare and production efficiency in dairy cow farming. Traditionally, identification methods such as ear tags, smart collars, and Radio-Frequency Identification (RFID) are commonly used in livestock farming [12]. These systems are suitable for indoor facilities and are typically associated with static infrastructure, such as milking, feeding, or watering units. However, innovative identification methods, such as image-based pattern/spot [13,14,15], nose prints [16,17], or head/face recognition systems [18], have emerged as alternatives to traditional systems. These new systems can provide more precise, cost-effective, and efficient monitoring and management of cows, and they are also suitable for deployment in mobile setups.

**Figure 1:**
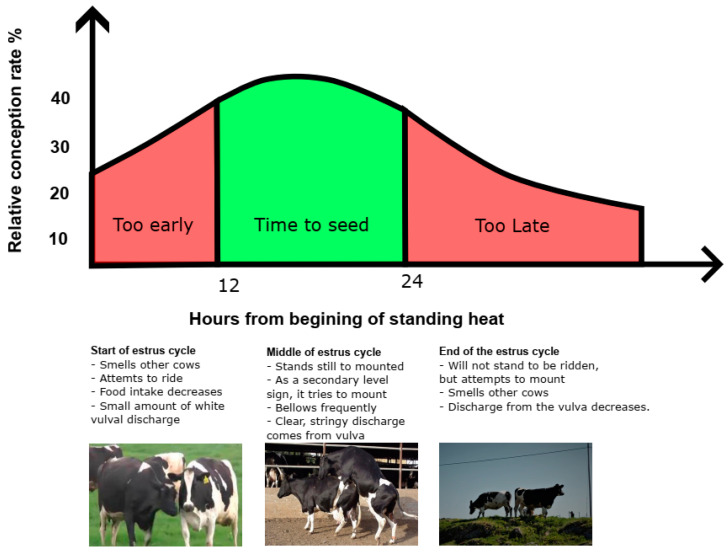
Estrus cycle diagram.

The estrus period in cows refers to the time when a mature cow is most fertile and ready for conception. This period is usually marked by specific movements and behaviors. Cows typically have an estrus cycle that lasts around 21 days, and if they do not become pregnant, they will enter another estrus period approximately 21 days later [19,20]. The estrus period of cows can vary depending on factors such as age, seasonal conditions, diet, etc. Monitoring estrus in cows is important to ensure that pregnancy occurs within a short period of time after giving birth. In Reith and Hoy’s classification of estrus signs, both primary and secondary signs of estrus are explained [21]. Primary signs include “standing to be mounted”, which is the most prominent behavior, indicating that cows are ready for mating during the estrus period. However, a decrease in the frequency of this behavior has been noted, especially in cows with high milk production. The duration of this behavior may be shorter in high-yielding cows. Secondary signs include mounting behavior, increased activity, changes in rumination time, agonistic interactions, and social interactions. Mounting behavior is a secondary sign that begins before the primary sign of estrus and continues afterward. The frequency of cows mounting each other or attempting to mount during the mating period can be considered a more reliable indicator for estrus detection. These signs are important for accurately detecting the estrus period and determining the optimal time for artificial insemination. Additionally, the text emphasizes the impact of environmental factors such as housing conditions, floor features, and climate on these signs. Observing the estrus period of a cow is important for optimal timing of insemination to increase productivity [19,22]. The estrus cycle seen in cows is shown in Figure 1. Artificial insemination can be performed between the 12th and 24th h of estrus, but the highest conception rate is achieved between the 12th and 18th h.

The detection of estrus using traditional methods involves employees on the farm monitoring the mounting behavior of the cows. Missing the estrus period due to any disruptions can result in economic losses for the business. These economic losses can lead to a reduced milk yield, delayed insemination by 21 days, and a one-month delay in calf birth [23,24,25,26]. For example, in a farm with 10 cows, if estrus is missed once for each cow, it causes one calf loss in a year in the number of calves that will be born on the farm.

Machine learning and deep learning techniques are among the latest technologies used to automate estrus detection. These techniques detect the estrus period based on the activities, behaviors, and/or physiological characteristics of the cows relying on their video images. This enables increased productivity in farming by preventing the need for employees to spend time on estrus detection and minimizing the risk of inaccurate detections [22].

The mounting behavior of the cow in estrus is shown in Figure 1. Memmedova and Keskin aimed to detect cows’ estrus by utilizing the movement characteristics of cows during the estrus period [23]. They aimed to detect estrus using a fuzzy logic model that includes features like the level of activity of a cow and the time elapsed since giving birth. The movement characteristics of the cows were measured by attaching step counters to their front legs. Memmedova and Keskin were able to detect the cows’ estrus state at a rate of 98% using the method they used [23].

Yıldız and Özgüven conducted a study that aimed to detect cows’ estrus by examining not only the movements displayed by cows during the estrus periods but also the effects of the season [27]. They collected movement data and seasonal information from 186 cows that exhibited estrus, of which 78 were dairy cows. They trained single-layer artificial neural network models on this data and obtained an estrus period detection accuracy of 97% [27]. Arago et al. developed a system that aims to detect cows’ estrus by tracking the mounting behavior displayed by cows during the estrus period [28]. In their study, they trained two artificial neural network models using the Tensor Flow Object Detection Application Programming Interface (API) with the goal of detecting the estrus event within 100 m. They carried out the detection process with the trained models by analyzing images taken from cameras installed at specific angles. The system they developed has an accuracy rate of 94%. In addition to these academic studies, there are also products that detect estrus in cows. Actimoo is a commercial estrus tracking system [29]. This product detects estrus based on physical data collected by an activity meter attached to the neck of the cows, and its accuracy rate is defined as 80%. Another product used for estrus tracking is the estrus band. Estrotect bands are attached to the backs of cows, and they change color during the estrus period when another cow mounts the band-wearing cows, indicating estrus [30].

Various methods exist for detecting the estrus period in cows used in production. These methods typically involve attaching a pedometer-like collar or wearable bracelet device [23,27,29,31,32,33] to the cow or applying painting patches called estrus patches [30] to the tail region of the cows. The main disadvantage of commercial wearable devices used in animal husbandry, namely wristbands, collars, or paint patches, compared to computer vision-based systems, is the necessity of allocating a device to each animal and, therefore, pricing per animal. In addition, painting patches, such as Estrotect [30], are disposable, although they are cheap and practical. Actimoo [29] and SCR [33], which are commercial systems, have a limited usage time (as long as battery life) and require infrastructure because they communicate wirelessly with the intermediary device; that is, they are environment dependent.

Systems supported by deep learning, which could be considered more recent, are still in the research stage, and a commercially matured system has not been encountered. Existing visual systems serve a single purpose, such as estrus detection. This paper proposes the development of a system that sequentially performs both estrus detection and cow identification processes for use in augmented reality applications in dairy cows. The system we propose is not individual based but refers to a volume such as a room, cow pen, or open area, and its mobility is higher, especially for use in devices such as smartphones or drones. The method proposed in this study aims to contribute to the following aspects:

- Introduce a deep learning-based method to visually identify animals on a livestock farm;
- Introduce a deep learning-based method for detecting standing mounted and mounting behaviors, the primary and secondary signs of estrus behavior. This brings new technology to the dynamic structure of modern animal husbandry;
- Present a high-accuracy system by integrating estrus detection and cow identification processes through the proposed method.

## 2. Materials and Methods

**Figure 2:**
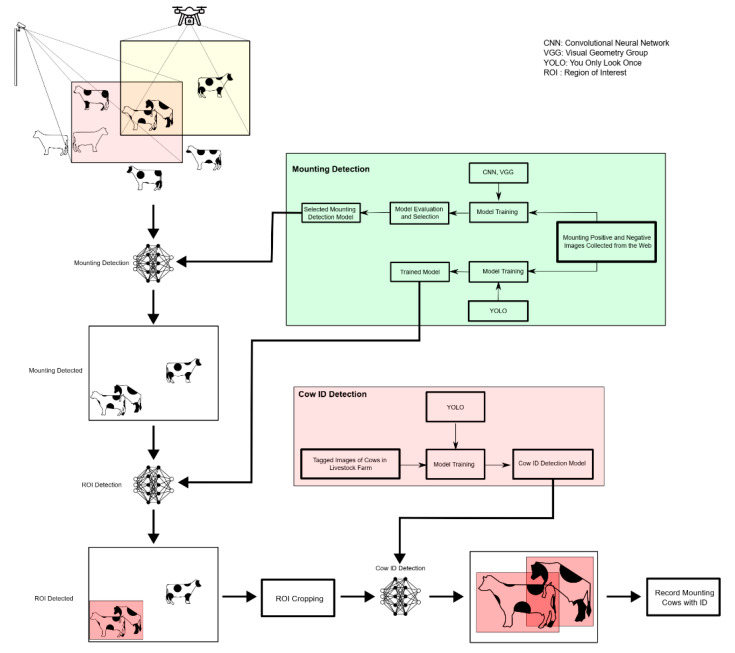
General method.

The core idea of the method is shown in Figure 2. The general structure of the method involves the sequential utilization of two deep learning-based detectors for estrus and cow identification, as illustrated in Figure 2. In the general method, the mounting detection process is initially performed using a CNN or VGG, followed by determining the region of interest (ROI) where the mounting action occurs using YOLO. After identifying the ROI, it is cropped with a padding of 20 pixels around it. Cow ID detection is then carried out on this cropped ROI using YOLO. Subsequently, the cow IDs are registered in the system. The details of these procedures are presented in the subsequent subsections.

The estrus detector operates by transferring a model trained on images containing positive and negative cases of estrus periods, collected from the internet to real videos. This method was chosen due to the labor-intensive and time-consuming nature of monitoring and photographing the estrus period. For cow identification, a dataset of images was gathered from various angles of cows present in the livestock facility and manually labeled. The obtained dataset was utilized for this purpose. The two models obtained were tested on images captured from drones, smartphones, and pan-tilt cameras. Initially, estrus detection is performed, followed by the identification of cows during the estrus period, enabling labeling within the facility.

### 2.1. Dataset and Transfer Learning for Mounting Detection

After the first calving, a dairy cow can be counted in the productive stage [34]. The lifecycle in this stage is a sequence of lactation (up to 305 days), dry period (about 60 days), and calving (about 280 days) [34,35,36]. Even if samples are collected in an area with many animals due to the fact that obtaining comprehensive data from different animals would take a year and necessitate continuous observation by an individual who tags these moments through video, it has been decided that the process of data collection is both laborious and time consuming. To alleviate the burden on human resources, expedite the process, and expand the dataset, we opted to source images featuring both mounting and non-mounting behavior from the internet. Our research is centered around a dataset comprising cows of diverse breeds, specifically Simmental, Holstein, Jersey, and Brown Swiss (Montofon). This dataset encompasses images of cows engaged in mounting behavior and those not involved in such activities, all captured from various angles. The dataset was curated by collating images from online sources, specifically from search engines where cow images are publicly shared. Importantly, each image underwent a manual labeling process to categorize them appropriately.

**Figure 3:**
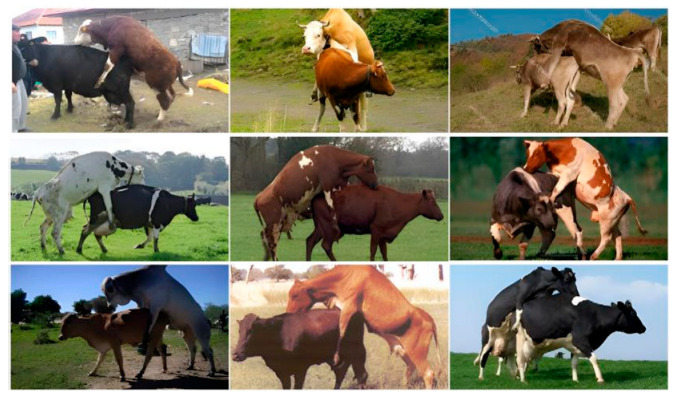
Dataset positive class image collage.

**Figure 4:**
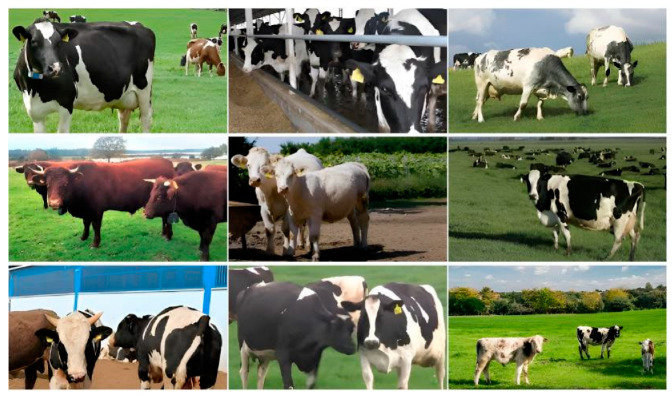
Dataset negative class image collage.

The dataset was enriched with data augmentation techniques to prevent the models from overfitting. During the data augmentation phase, techniques such as rotating images by a specific angle and zooming in and out were benefited [37]. The total size of the dataset is 1638. The test data size is 492 (30%), and the training size is 1146 (70%). The distribution is not stratified. The two-class dataset consists of a total of 1638 images, comprising 937 images of cows in estrus and 701 images of cows not in estrus. Figure 3 and Figure 4 display some examples belonging to the positive and negative classes within the dataset. The images in the dataset were preprocessed and normalized prior to training. During the preprocessing stage, images with different pixel dimensions were resized to (224,224) pixel dimensions.

### 2.2. Dataset and Cow Identification

The dataset was collected from a farm in the Aydın region, and this cow recognition project was enhanced through the use of drone technology. All 300 dairy cows in the full-capacity section of the farm were captured in high-quality images, which were then analyzed using artificial intelligence techniques. The inclusion of cows from different breeds, such as Holstein and Simmental, highlights the ability of artificial intelligence and deep learning to accurately recognize various breeds and characteristics.

### 2.3. Deep Learning Architectures

#### 2.3.1. Convolutional Neural Network (CNN)

**Figure 5:**
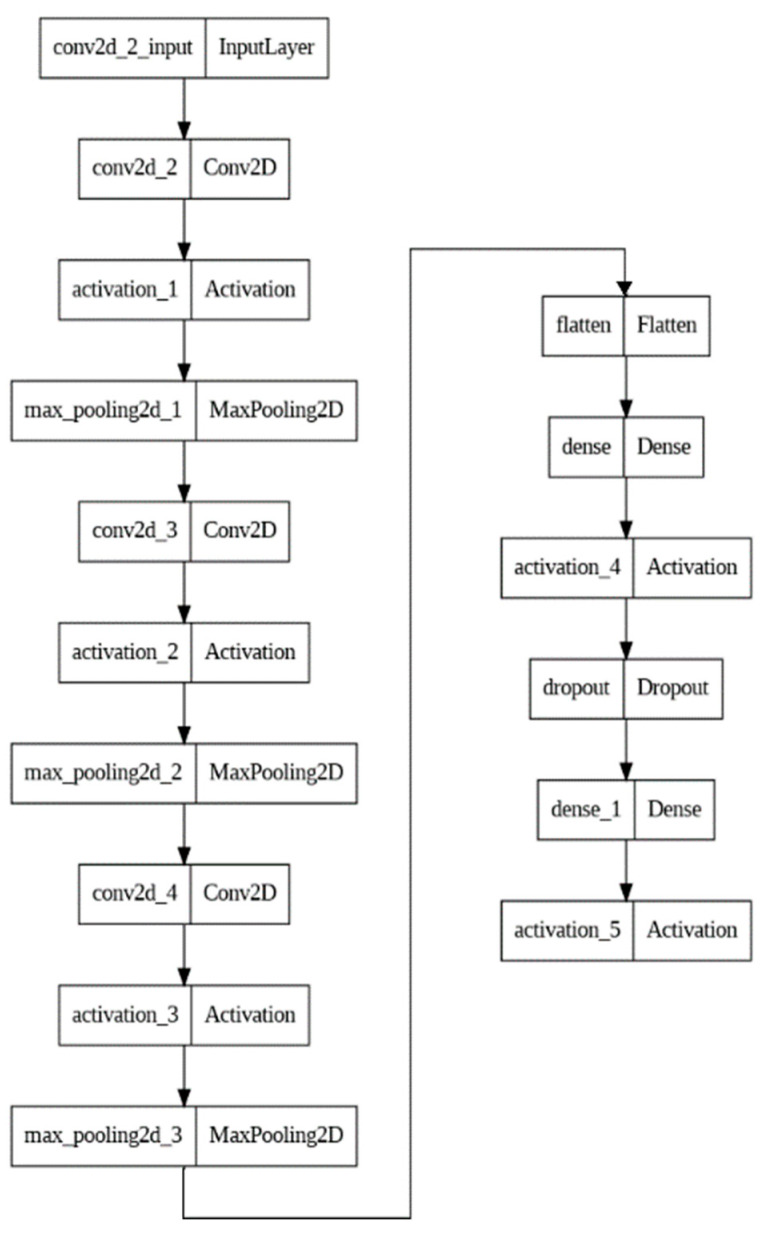
Proposed CNN architecture.

A CNN was used only for mounting detection purposes; therefore, only the estrus dataset consisting of images of mounting or non-mounting situations was used, and the images were collected from the internet. Artificial neural networks are models that are based on the functioning of the human brain. The goal of this structure is to perform the learning process, interpret the acquired knowledge, and make decisions autonomously. Convolutional neural networks (CNNs) are a type of artificial neural network that are used primarily for image recognition and computer vision tasks, although they can also be used for other types of data processing, such as natural language processing. CNNs have revolutionized the field of computer vision and continue to be an active area of research and development [38,39]. Computers must recognize and convert incoming images into a computationally manageable matrix format. The first layer in a CNN is a convolutional layer, which applies a set of filters to the input image to extract features, such as edges and corners. The output of the convolutional layer is then passed through an activation function to introduce non-linearity into the model. The output of the activation function is then passed through a pooling layer, which reduces the dimensionality of the feature maps while retaining the most important information. The final output of the network is typically a fully connected layer that performs classification. It learns the impact of these differences on the label during the training phase and then uses this knowledge to make predictions for new images. In this study, a 9-layer convolutional neural network was used, as seen in Figure 5, and the network was trained for 20 epochs with binary classification.

#### 2.3.2. VGG-19

**Figure 6:**
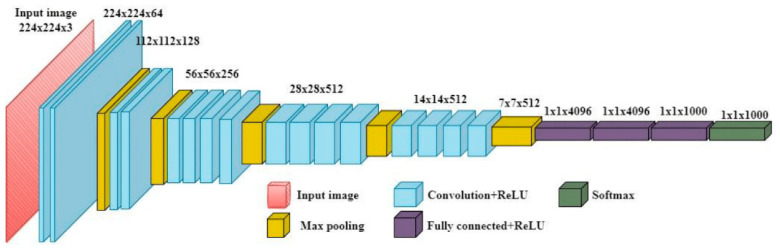
VGG model’s architecture.

VGG-19 was used only for mounting detection purposes; therefore, only the estrus dataset consisting of images of mounting or non-mounting situations was used, and the images were collected from the internet. The VGG-19 is a CNN architecture that was introduced by the Visual Geometry Group (VGG) at the University of Oxford [40]. It is a deep CNN with 19 layers that was designed primarily for image classification tasks. The VGG-19 architecture consists of a series of convolutional layers with 3 × 3 filters, followed by max pooling layers and rectified linear unit activation functions. The convolutional layers are organized into five blocks, with each block containing multiple convolutional layers and a max pooling layer. The final layers of the VGG-19 architecture consist of fully connected layers that perform the classification task. The output of the last fully connected layer is fed into a SoftMax activation function to produce the class probabilities [40,41]. In this study, as seen in Figure 6, we removed the fully connected layer of the pretrained VGG-19 model and added a new connection layer based on the number of classes in the dataset.

#### 2.3.3. YOLO

The YOLOv5 method was used for both mounting regions of interest detection and cow identification. For this reason, two models were created separately for each dataset. YOLOv5 is a convolutional neural network (CNN) architecture that was introduced in 2020 as an evolution of the popular YOLO (You Only Look Once) object detection model [42]. YOLOv5 is designed primarily for real-time object detection and recognition tasks, including the detection of people, vehicles, and animals. Its acronym stands for ”You Only Look Once”, referring to its ability to quickly and efficiently make object detection predictions in a single step. YOLO divides the input image into an N × N grid, and each grid cell determines the presence of an object within its area, considering the object’s center. The grid cell that determines the center of the object will also find the class, height, and width of the object and draw a bounding box around it [43]. This simplifies the architecture and improves speed and accuracy. In our study, the dataset was labeled as positive or negative, and the YOLOV5 model, which is the 5th version of YOLO, was trained on this dataset for 150 epochs.

### 2.4. Deep Learning Performance Evaluation and Model Selection

The hyperparameters of the algorithms used in the study, the optimizer used, the preferred primary performance metric, train test dataset ratios, and loss value are shared in Table 1. Models are focused on a binary classification problem. Models are compiled with the “binary_crossentropy” loss function and “rmsprop” optimizer presented in Table 1. The “accuracy” metric is used to evaluate the model’s performance in terms of accuracy. To measure classification performance, accuracy, F1 score, precision, and recall performance metrics, as presented in Table 2, are used. The dataset was split into 70% training data and 30% test data. In our study, we opted not to use k-fold validation for several reasons. Firstly, k-fold validation may present challenges during deployment, as determining which fold to use in real-world scenarios lacks a clear criterion. Additionally, our models demonstrated high performance without the need for extensive hyperparameter tuning, making the application of k-fold validation less crucial in our context. Moreover, the computational cost associated with k-fold cross-validation, which requires training a separate model for each fold, was deemed excessive given the satisfactory performance of our models. In summary, our decision aligns with practical considerations related to deployment, a lack of hyperparameter tuning needs, and the desire to maintain computational efficiency in the field of computer engineering.

## 3. Results and Discussion

The dataset containing images of cows’ mounting movements during the estrus period was used to successfully detect estrus in cows. Three different deep learning models, namely the convolutional neural network, YOLO, and VGG-19, were trained on the dataset.

### 3.1. Estrus Detection

Performance metric results obtained by the methods used for estrus detection are presented in Table 3. Accordingly, the highest accuracy value was obtained with VGG-19 and is 99%. The findings obtained with each method are discussed in the subsections.

#### 3.1.1. Mounting Detection with CNN

**Figure 7:**
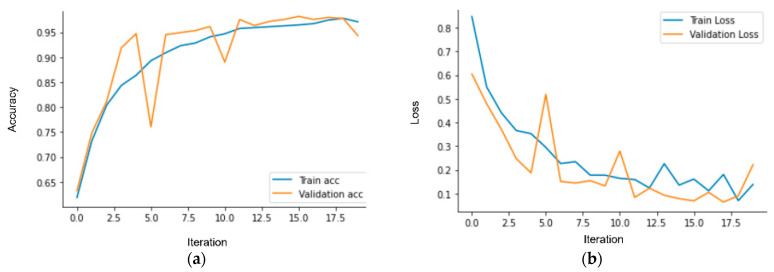
CNN training and test: (**a**) accuracy and (**b**) loss.

**Figure 8:**
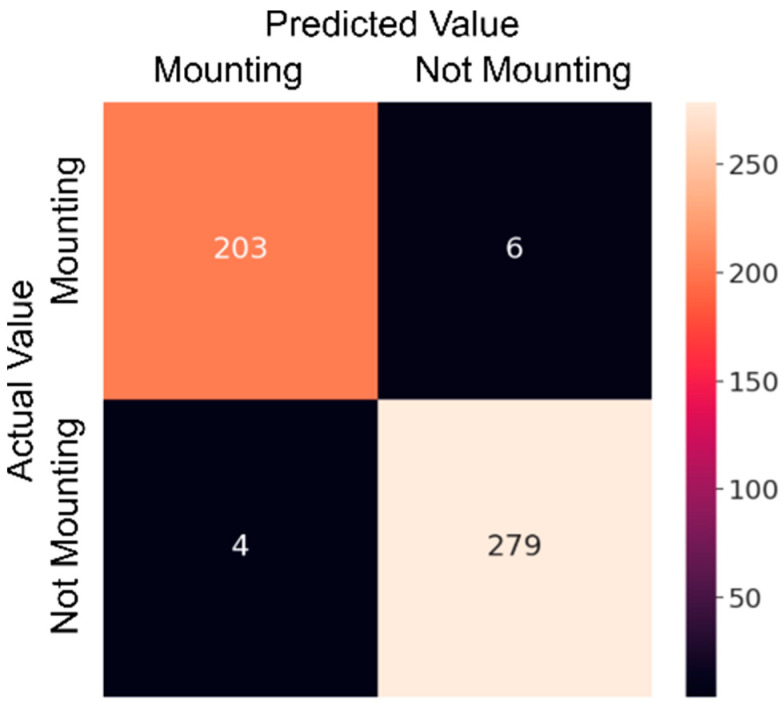
The CNN model’s confusion matrix of the test dataset.

**Figure 9:**
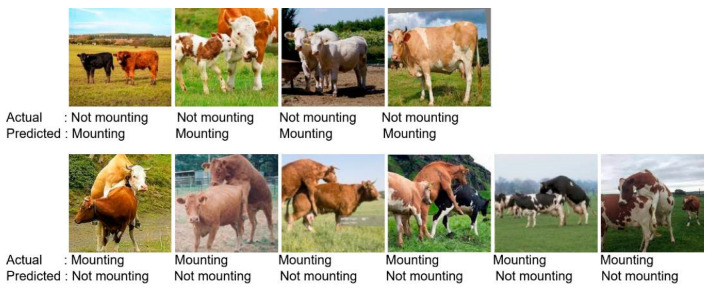
CNN false predictions of the test dataset.

The CNN model, which is a classical approach to image recognition problems, has been developed for this study. The developed CNN model consists of nine layers, and the dataset has been divided into 70% for training and 30% for testing. The CNN training process consists of 20 epochs, with a duration of 25.2 min. While training the CNN model, the GPU services of Google Colab, specifically the T4 GPU, were utilized. In this process, using 32 GB of RAM proved to be sufficient. After training on the training dataset for 20 epochs, Figure 7 shows that the model achieved an accuracy rate of 98%, as represented in Table 3. The model’s loss value is calculated to be 0.1. Interpreting the CNN results requires an understanding of the metrics used to measure the model’s performance, consideration of the dataset and training parameters used, and careful analysis of the results. Figure 8 presents a confusion matrix, indicating that the CNN model correctly identified 203 out of 207 estrus cases in the test dataset. In the negative cases where the cows did not show estrus, it accurately detected 279 out of 285 cases. Figure 9 includes an example of prediction results from the trained model. It is observed that the second case is the false predictions, and this error seems to stem from the model perceiving the size difference between cows and calves as a feature. In Figure 9, it is observed that errors occur in images predicted as false not mounting when the feet are aligned, in contact, or very close. In images predicted as false mounting, incorrect results are noticeable in crowded situations. However, despite all these errors, only 10 out of a total of 492 test images have been predicted as false.

#### 3.1.2. Mounting Detection with VGG-19

**Figure 10:**
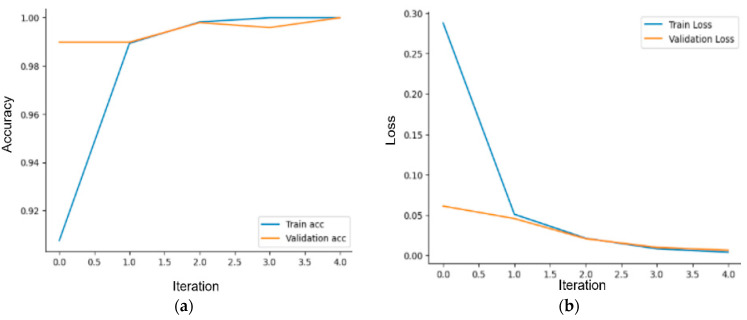
VGG-19 training and test: (**a**) accuracy and (**b**) loss.

**Figure 11:**
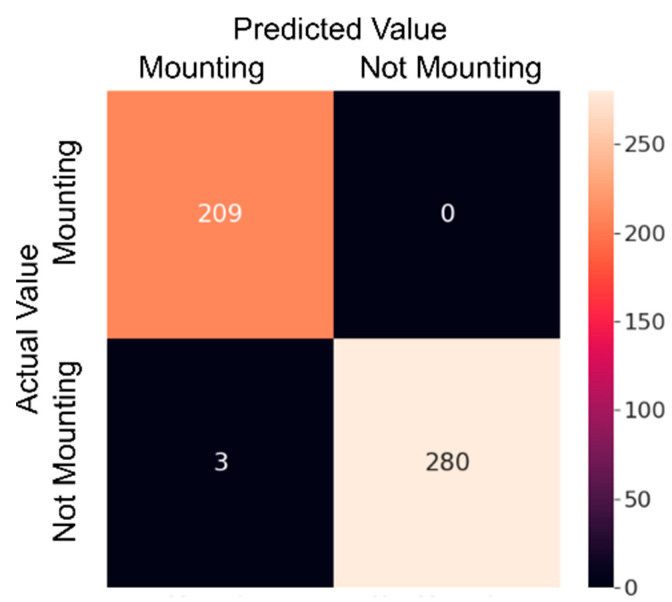
VGG-19 model’s confusion matrix of the test dataset.

**Figure 12:**
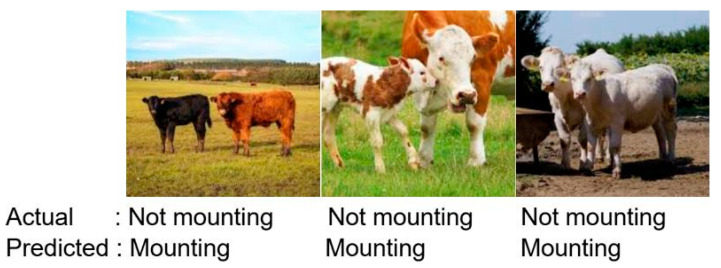
VGG-19 model false predictions of the test dataset.

The VGG-19 model, which is a transfer learning model, has been trained on the dataset. The dataset has been divided into 70% for training and 30% for testing. Considering the two classes in the dataset, the fully connected layer has been adjusted accordingly, and the model has been trained on the training dataset for 20 epochs with a duration of 28.3 min. While training the VGG-19 model, the GPU services of Google Colab, specifically the T4 GPU, were utilized. In this process, using 32 GB of RAM proved to be sufficient. The interpretation of the VGG model results may vary depending on the task and performance criteria for which the model is used. The VGG model is commonly used for image classification tasks, and accuracy is the most common performance measure for this task. Therefore, the VGG model results are typically presented in the form of a table or graph with high accuracy, indicating that the model is successful in the classification task with high accuracy. The trained model achieved an accuracy rate of 99%, making it the most successful model in our study. Furthermore, the developed model correctly identified 280 out of 283 negative states. Figure 10 shows that the developed model’s loss value approaches zero. Figure 11 displays a confusion matrix, which demonstrates that the developed VGG-19 model successfully predicted 209 out of 209 estrus states in the test dataset. Figure 12 includes an example of prediction results from the trained model. The origin of all false predictions made by the VGG-19 model is attributed to the data augmentation techniques applied to prevent overfitting. Due to operations, such as rotation, zooming, and others applied within the dataset, pixel losses occurred, leading to erroneous classifications by our transfer learning model. Similar to the CNN model, in images predicted as false negatives in Figure 12, errors are observed in aligning the feet in close or touching images. However, in the VGG model, there are no images predicted as false mounting, and only three out of 492 test images are falsely predicted.

#### 3.1.3. Mounting Region of Interest Detection with YOLOv5

**Figure 13:**
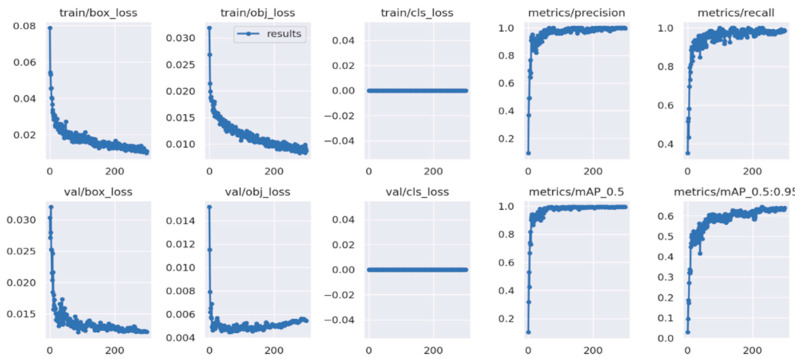
YOLOv5 accuracy.

**Figure 14:**
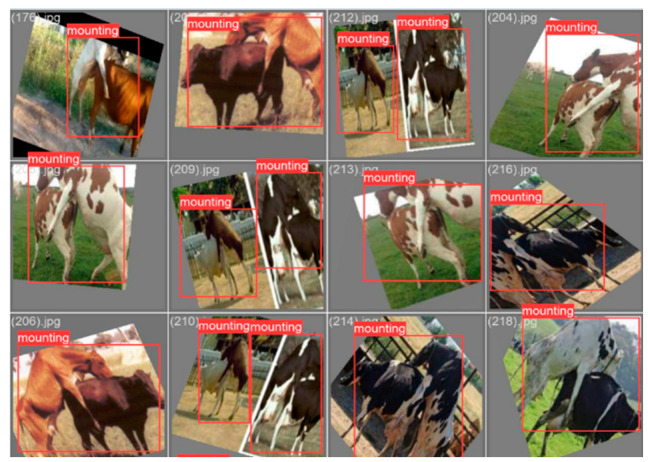
YOLO model prediction examples.

The YOLOv5 deep learning model was used to detect estrus states by labeling the 937 images in the dataset that show estrus. YOLOv5 can be evaluated using a variety of performance metrics, and these include mean average precision (mAP), accuracy, precision, and recall. mAP is a widely used metric to examine the results of object recognition models. The higher the map of an object recognition model, the more accurate and reliable the model is. Accuracy measures the evaluation of samples, showing that the modeling is correct. Based on Figure 13, the trained YOLOv5 model achieved a 98% accuracy rate in detecting estrus conditions. The “metrics/mAP 0.5” value in this figure shows the accuracy rate of our model. The “loss” values in the figure indicate how many errors the model made during its training. Our model continued its training until we minimized our loss values. The YOLOv5 training process is 150 epochs, and the duration is 32.4 min. While training the YOLO model, the GPU services of Google Colab, specifically the T4 GPU, were utilized. In this process, using 32 GB of RAM proved to be sufficient. Figure 14 includes an example of prediction results from the trained model.

### 3.2. Cow Identification

**Figure 15:**
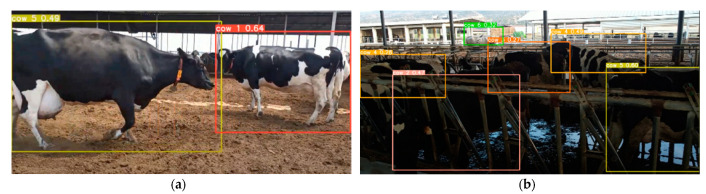
YOLO cow identification: (**a**) cow identification from a pan-tilt camera; (**b**) cow identification from a drone camera.

**Figure 16:**
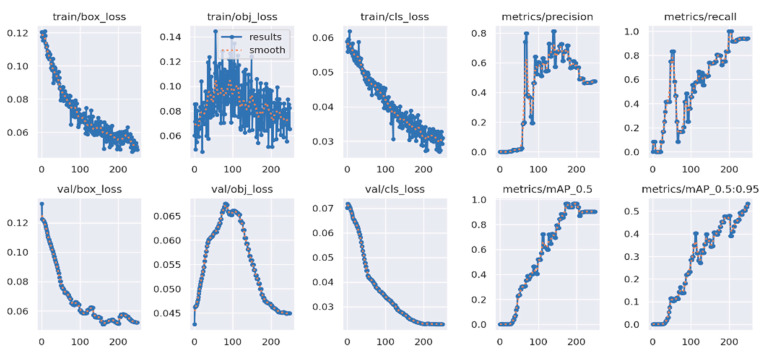
YOLO cow identification evaluation.

All 300 dairy cows in the full-capacity section of a farm in the Aydın region were captured with high-quality images. Sample images obtained for YOLOv5 used are presented in Figure 15, and evaluation results are presented in Figure 16. The YOLOv5 training process is 150 epochs, and the duration is 15.2 min. The cow identification accuracy of the system is about 95%.

### 3.3. Cascaded System Results

**Figure 17:**
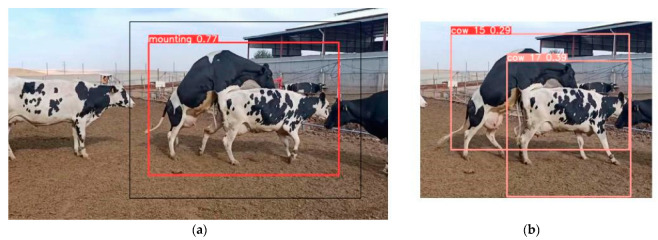
Cascaded system results: (**a**) mounting detection and (**b**) cow identification after from mounting detection crop.

The process of cow identification is illustrated in Figure 17, where cropping is performed around the relevant bounding box after mounting detection, with a 20-pixel padding. Primary and secondary behavioral signs for estrus, such as waiting periods for standing to be mounted and mounting, are depicted in Figure 17a. Subsequently, the identified identifiers for both cows are presented in Figure 17b. Following this detection and animal marking process, relevant information can be sent, and the artificial insemination process can be initiated. Mounting couple detection accuracy is 94%.

### 3.4. Comparison with Similar Studies

Table 4 illustrates the differences between our approach and other common methods. Commercial systems in the literature are designed as wearable smart collars and paint patches, as presented in Table 4. Determining the onset of estrus, which is considered the most suitable time for the beginning of cows’ reproductive cycles, and artificial insemination, supports livestock farming that meets a large portion of the increasing country’s population’s food needs. On the other hand, it will also save the farm owner from the delays and economic losses that occur during these reproductive cycles. If the farm owner misses or incorrectly detects the estrus period, they may face problems such as a loss in milk production, a calf born with a delay of at least a month, and the inability to take advantage of government support. The purpose of our study is to detect the estrus periods and thereby help farm owners avoid economic losses and delays. Traditional methods used to detect estrus in cows include observing physical movements. Among these studies, Memedova and Keskin achieved 98% accuracy in detecting estrus by tracking the physical movements of cows with a fuzzy logic model that they developed [23]. In another study, which was prepared as a doctoral thesis by Yildiz, an artificial neural network model was developed that used not only physical movements but also seasonal data, achieving 97% accuracy [27]. Arago et al. aimed to detect cows in estrus that display mounting behavior, using models trained on images of cows [28]. Although the collected dataset has not been shared, a system that works with a 94% accuracy rate was developed with the help of the trained model. Our study and the results of other studies conducted in the literature are shown in Table 5.

Behaviors such as standing to be mounted and mounting are all just symptoms of the estrus period, and as shown in Figure 1. If these symptoms are detected, only the success rate of artificial insemination increases [21]. While livestock wearables equipped with IMU, painting patches, and visual computing systems successfully identify mounting and/or standing-to-be-mounted behaviors, they are commonly referred to as estrus detection systems [28,29,30,31,32,33]. The proposed method is a visual system and is used to detect mounting and/or standing-to-be-mounted behaviors. In this way, it is possible to increase the success rate of artificial insemination by identifying only animals that are likely to be in estrus. It is important to mention that false estrus warnings, resulting from social mounting interactions in dairy cows, can also be detected by the system. This limitation has implications for the broader impact of smart estrus detection studies presented in Table 4 as well. In addition, the interactions creating false alerts are short in duration [21,44]. Therefore, it is possible to reduce false alerts by separating longer-term standing-to-be-mounted situations and creating a rule-based system for when the visual system is triggered. If it is desired to determine the exact status of the cows rather than predicting, more comprehensive studies are required that include not only visual or biomechanical data but also physiological data and veterinary examination reports.

**Table 4:** Comparison of the systems to detect the estrus period and cow identification.

Reference	Sensor	Estrus Detection	Cow Identification	Cost	Lifetime
Actimoo [29]	IMU	Pattern recognition from IMU signals obtained from cow collars	Smart collar matching with the ID of the dairy cow	~EUR 120 per cow	5 years battery life
SCR Heatime [33]	IMU	Pattern recognition from IMU signals obtained from cow collars	Smart collar matching with the ID of the dairy cow	~EUR 200 per cow	7 years battery life
Estrotect [30]	Paint	Patch that changes color when the cow mounts	Accomplished by seeing the painted cow by the farmer	EUR 2.5 per usage	Disposable
[28]	PTZ Camera	Faster R-CNN and SSD cow localization and tracking	NA	NA	No battery
[45]	Camera	Detection of cow images with deep learning (YOLOv5)	NA	NA	No battery
[46]	Multiple cameras	Detection of ewe images with deep learning (YOLOv3)	NA	NA	No battery
[47]	RFID	NA	Yes	Under EUR 1 per cow	No battery
[48]	IMU and RFID	NA	Yes	Under EUR 20 per cow	Rechargeable Li-Po (728 days)
Proposed method	Camera	Detection of cow images with deep learning	Classification of images with deep learning	NA	No battery

**Table 5:** Comparison of machine learning-based methods in estrus detection and cow identification.

Reference	Sensor	Software	Estrus Detection Accuracy (%)	Cow Identification Accuracy (%)
[23]	IMU	Fuzzy logic model	98	NA
[27]	IMU	Deep learning	97	NA
[29]	IMU	NA	80	NA
[28]	Camera	Deep learning	94	NA
[45]	Camera	Detection of cow images with Deep learning (YOLOv5)	94.3	NA
[49]	Camera	Computer vision	90.9	NA
[50]	Camera	YOLOv3	82.1	NA
[51]	Camera and ear tag	CNN	NA	84
[52]	Camera	YOLO and faster R-CNN	NA	84.4
Proposed CNN model	Camera	Deep learning	98	NA
Proposed VGG-19 model	Camera	Deep learning	99	NA
Proposed YOLO model	Camera	Deep learning	98	95

## 4. Conclusions and Future Works

This manuscript introduces an innovative approach utilizing machine learning for the identification of individual cows and the detection of estrus behaviors. While previous studies have successfully detected estrus behaviors through machine vision, identifying individual cows in estrus within a herd has proven challenging. This pursuit is deemed valuable, and the development of an algorithm capable of providing such information holds significant importance. In livestock production, various methods exist for detecting the estrus period, such as attaching pedometer-like devices or applying estrus patches. However, these commercial wearable devices pose limitations, including the need for one device per animal, pricing concerns, environmental dependency, and restricted lifespans. This study proposes a deep learning-based system for both estrus detection and cow identification, addressing the shortcomings of existing methods and aiming to contribute to automated livestock management.

The proposed approach seamlessly integrates estrus period detection and cow identification using AR. The process commences with deep learning-based mounting detection, followed by the identification of the mounting ROI through YOLOv5. Subsequently, the ROI is cropped with padding, and cow ID detection is performed using YOLOv5 on the cropped ROI. The system then records the identified cow IDs. Demonstrating exceptional accuracy, the proposed system achieves 99% precision in detecting mounting behavior, 98% accuracy in ROI identification for mounting, and 94% accuracy in detecting the mounting couple. The overall success of these operations underscores the potential of the proposed system in contributing to AR and AI applications within the realm of livestock farming. This research holds significance for automating livestock management through advanced augmented reality systems, showcasing efficiency in estrus period detection and cow identification by integrating CNN and VGG-16 detectors sequentially. This approach addresses the limitations of labor-intensive and time-consuming traditional monitoring methods.

Recognizing the critical importance of accurately determining the reproductive cycles of cows for sustainable livestock farming, this study emphasizes the economic benefits and increased production efficiency that result from informed decision making. By combining augmented reality and deep learning models, the research represents a scientific advancement, offering a non-intrusive alternative to traditional wearable systems. The proposed system also presents cost-effective and durable solutions for veterinary training in large businesses, with potential applications in pasture farming, drone adaptation, and even aspects of robotic shepherding. This study anticipates a positive impact on livestock management by providing a state-of-the-art solution that facilitates automation and enhances productivity in the livestock sector.

In conclusion, this research proposes a cutting-edge solution to enhance livestock management, offering a method that can detect estrus periods in an efficient way. The image-based approach for estrus period detection contributes to the literature and is poised to drive automation and productivity improvements in the livestock sector.

## Figures and Tables

**Table 1:** The model’s hyperparameters.

Model	Loss Function	Optimizer	Performance Metric	Train Data (%)	Validation Data (%)	Epoch	Mini-Batch Size	Learning Rate
CNN	Binary crossentropy	Rmsprop	Accuracy	70	30	20	32	0.001
VGG-19	Binary crossentropy	Rmsprop	Accuracy	70	30	20	32	0.001
YOLO	Binary crossentropy	Rmsprop	Accuracy	70	30	150	16	0.001

**Table 2:** Classification performance metrics.

Metric	Equation
Accuracy	$A=\frac{Number\;of\;True\;Positives+Number\;of\;True\;Negatives}{Number\;of\;Samples}$
Precision	$P=\frac{Number\;of\;True\;Positives}{Number\;of\;True\;Positives+Number\;of\;False\;Positives}$
Recall	$R=\frac{Number\;of\;True\;Positives}{Number\;of\;True\;Positives+Number\;of\;False\;Negatives}$
F1 Score	$F1=\frac{2\times P\times R\;}{P+R}$

**Table 3:** Performance metric results of the models.

Model	Accuracy (%)	Precision (%)	Recall (%)	F1 Score (%)
CNN	98	97	98	97
VGG-19	99	99	98	99
YOLO	98	98	98	97

## Data Availability

Please contact the corresponding author for the findings, model, and dataset obtained in the study.
